# The Ditch and the House. Megara Hyblaia by the early 7th c. BC.

**DOI:** 10.12688/openreseurope.20518.1

**Published:** 2025-07-14

**Authors:** Frédéric Mège, Eleonora Delpozzo

**Affiliations:** 1 Department of Humanities, Ca' Foscari University of Venice, Venice, Veneto, Italy

**Keywords:** Megara Hyblaia; Archaic Sicily; Ancient Construction; Architectural Energetics; 3D Modelling; BIM.

## Abstract

**Background:**

Megara Hyblaia, a Greek colony founded in the third quarter of the 8th century BC on Sicily’s eastern coast, exemplifies early Western Greek urbanism. The city flourished until 483 BC, when it was depopulated by Gelo of Syracuse. By the early 7th century BC—about two generations after its foundation—Megara had likely reached its maximum intramural size of approximately 60 hectares. This study focuses on that formative phase to evaluate whether the community possessed the resources to undertake two key construction projects: the first city fortification and residential expansion.

**Methods:**

Archaeological evidence shows that residential structures, initially limited to the agora, spread across the Southern Plateau and toward the Archaic Western Gate by the early 7th century BC. This urban spread correlates with the construction of the city’s earliest defensive structure, an “agger-wall” comprising a ditch, wall, and earthen bank. Though the exact chronology is uncertain, stratigraphic and architectural analysis places its construction early in the 7th century BC. The article investigates the feasibility of these projects using two case studies: the agger-wall and the house on plot 113W4, a well-preserved pastas-type dwelling dated to the first quarter of the 7th century. Using data from stratigraphic excavation, 3D modelling, Building Information Modelling (BIM), and architectural energetics, the study estimates construction time and labour requirements.

**Results:**

Findings suggest that the second-generation population could have built both the fortifications and sufficient housing within a year, without disrupting agriculture or essential tasks. The study also highlights the potential of digital tools in heritage research.

**Conclusions:**

This study evaluates the feasibility of early construction projects at Megara Hyblaia, revealing that 7th-century BC inhabitants likely had sufficient labor for fortifications and housing. It also highlights the promise and limitations of BIM tools in reconstructing ancient architecture and informing future digital heritage research.

## Introduction

**Figure 1.**
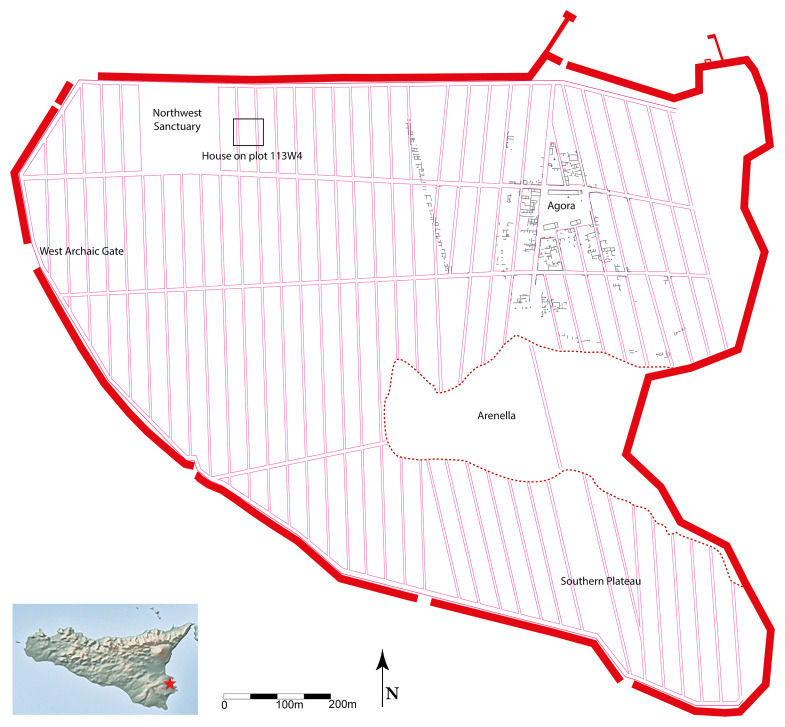
Schematic plan of archaic Megara Hyblaia.

Megara Hyblaia was part of the first wave of Greek expansion to the West, known as the Greek colonization. Founded in the third quarter of the 8th century BC on the eastern coast of Sicily ^1^, approximately 20 km north of Syracuse, the city flourished until 483 BC, when Gelo of Syracuse emptied the settlement and deported its inhabitants (Herod.7, 156). Before this upheaval, Megara Hyblaia had expanded to around 60 hectares, a spatial definition that may have been established as early as the first decades of the 7th century BC (see Figure 1).

This era is the one around which this paper revolves, namely: Megara Hyblaia, two generations after the time of its foundation. The earliest known houses date to the late 8th century BC and have been identified only around the Agora. Before this point, it remains undetermined how the first inhabitants sheltered, whether in perennial structures or in tents/huts ^2^. Be that as it may, by the mid-7th century BC, residential structures had spread throughout the city, from the agora district to the Southern Plateau and even to its westernmost areas, near the West Archaic Gate. As a matter of fact, houses from the first half of the 7th century are known around the Agora district, where they multiplied compared to the first phase of the late 8th century (estimates in De Angelis, 2003, p.40–45), but also on the Southern Plateau where they seem to appear right in the early 7th century ( Gras *et al.*, 2004, p.151–155). On the western end of the urban space, houses from the same era have been uncovered in the vicinity of the Archaic Western Gate ^3^. This demonstrates that, at this period, housing had stretched to the furthest limits of the intramural space, and this could well be related to the construction of the first defensive line of Megara.

These structures were first identified on the western limit of the city, before being investigated in its southern part, and then again on the western side ^4^. They belong to the “agger-wall” type, which includes, in the latter case, from the outside toward the inside: a ditch, a wall, and a bank. The bank is generally made of the material extracted when digging the ditch, while the wall serves the double purpose of a retaining wall and a fortification wall. The exact chronology for Megara’s agger-wall construction remains imprecise, but there is some body of evidence that allows us to realistically place it in the first half of the 7th century, most certainly around the beginning of the century (see note 5). In ancient cities, the creation of a city wall not only had a defensive purpose but also served to materialise the limits of the urban space (the intramural space, or *asty*).

Two generations or so after the foundation, we therefore witness the temporal and functional conjunction of a fortification that defined and protected the urban space, and of housing that projected itself away from the initial core and up to the furthest limits ^5^. The goal of the present article is to estimate whether the Megarian community at that time had the necessary resources to bear the costs of these construction projects—in other words, whether it had the required workforce and time ^6^. To that end, we will consider two case studies: the first fortification (the agger-wall) and the house on plot 113W4. The latter is the first to have been extensively excavated and recorded according to the strict requirements of stratigraphic archaeology: we therefore possess first-hand and accurate data on the construction technique and the chronology of the house ^7^. Located close to the Northwest sanctuary, the first phase can be dated to the first quarter of the 7th century, and thus belonged to the housing expansion to the western area. The ground plan was of the *pastas*-type—a design that tended to become canonical in the cities of Greek Sicily from this time onward ( Fusaro, 1982). The agger-wall was also well investigated and its structure accurately characterised, even if the precise dimensions of the ditch still remain somewhat conjectural, not having been fully excavated. To quantify these costs and their implications for the Megarian community, we will appeal to specific methods of investigation: a dedicated digital environment, 3D modelling and Building Information Modelling, and the field of architectural energetics.

## 3D modelling and BIM for archaeological reconstructions

In recent years, archaeology has become increasingly oriented toward a three-dimensional approach, as 3D technologies are now widely employed to record and analyse archaeological sites and monuments. The availability of this digital documentation enables the development of virtual reconstructions with a high degree of accuracy and scientific rigor, grounded in interpretation and comparative analysis. While these reconstructions are often disseminated primarily for communication and visualization purposes, they also hold significant potential for advancing archaeological research. Moreover, as these reconstructions are based on data obtained through precise digital survey methods, they can also be applied to fields such as architectural energetics, particularly for quantifying the volumetric distribution of construction materials in a given structure ( Lancaster, 2021).

The increasing use of 3D reconstructions has prompted the experimental application of Building Information Modelling (BIM) ^8^ within archaeology—not merely as a modelling tool, but as a potential methodological framework for data management, interpretation, and long-term conservation. Various efforts have been made to apply BIM to archaeological sites; however, preliminary results reveal a wide range of approaches to the subject ^9^. To date, only a limited number of projects have utilised BIM to create virtual reconstructions of lost buildings, based on historical and archaeological data, while the majority focus on modelling archaeological remains still *in situ* ( Garagnani *et al.*, 2016; Guerrero Vega & Pizzo, 2021). In this context, Megara Hyblaia represented a significant case study through which to explore the potential of BIM as a methodological framework and to experiment with its integration into architectural energetics research. The house on plot 113W4 was modelled using a BIM approach, whereas a more conventional 3D reconstruction was employed to visualize part of the fortifications and their building technique.

### Software framework

**Figure 2.**
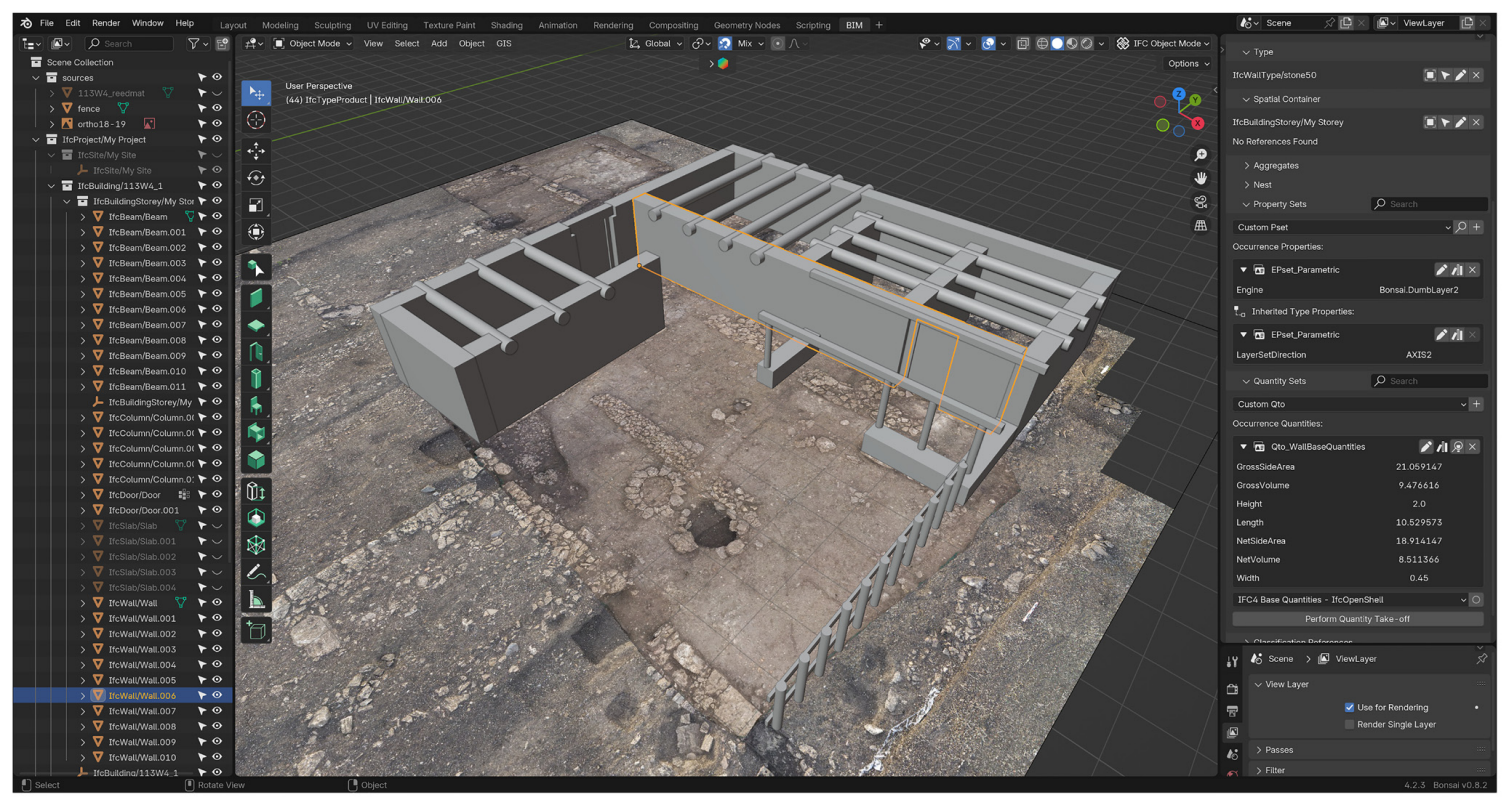
Screenshot of the software interface, showing Bonsai operating within Blender.

The field of 3D modelling encompasses a wide array of software solutions, each with distinct strengths and areas of application. Among these, Blender is widely recognised as one of the most versatile and commonly used open-source platforms for 3D modelling ^10^. In this regard, an open-source approach to BIM offers several key advantages: it enables the creation of native OpenBIM environments, supports vendor-neutral development, and promotes digital sustainability. Furthermore, it allows for the construction of workflows based on open standards such as IFC (Industry Foundation Classes) ^11^, which enhance interoperability and data longevity. For this project, Blender was employed alongside *Bonsai* ^12^, an open-source add-on for BIM modelling currently under development by the Open-Source Architecture community. *Bonsai* is designed to be a comprehensive and native IFC authoring platform, integrating BIM functionalities within Blender (see Figure 2).

### 3D modelling workflow

Each case study was modelled separately within Blender given the unique methodological approach required for each. In both cases, the virtual reconstruction was informed by excavation data, which provided the precise measurements and details necessary to the modelling process.

**Figure 3.**
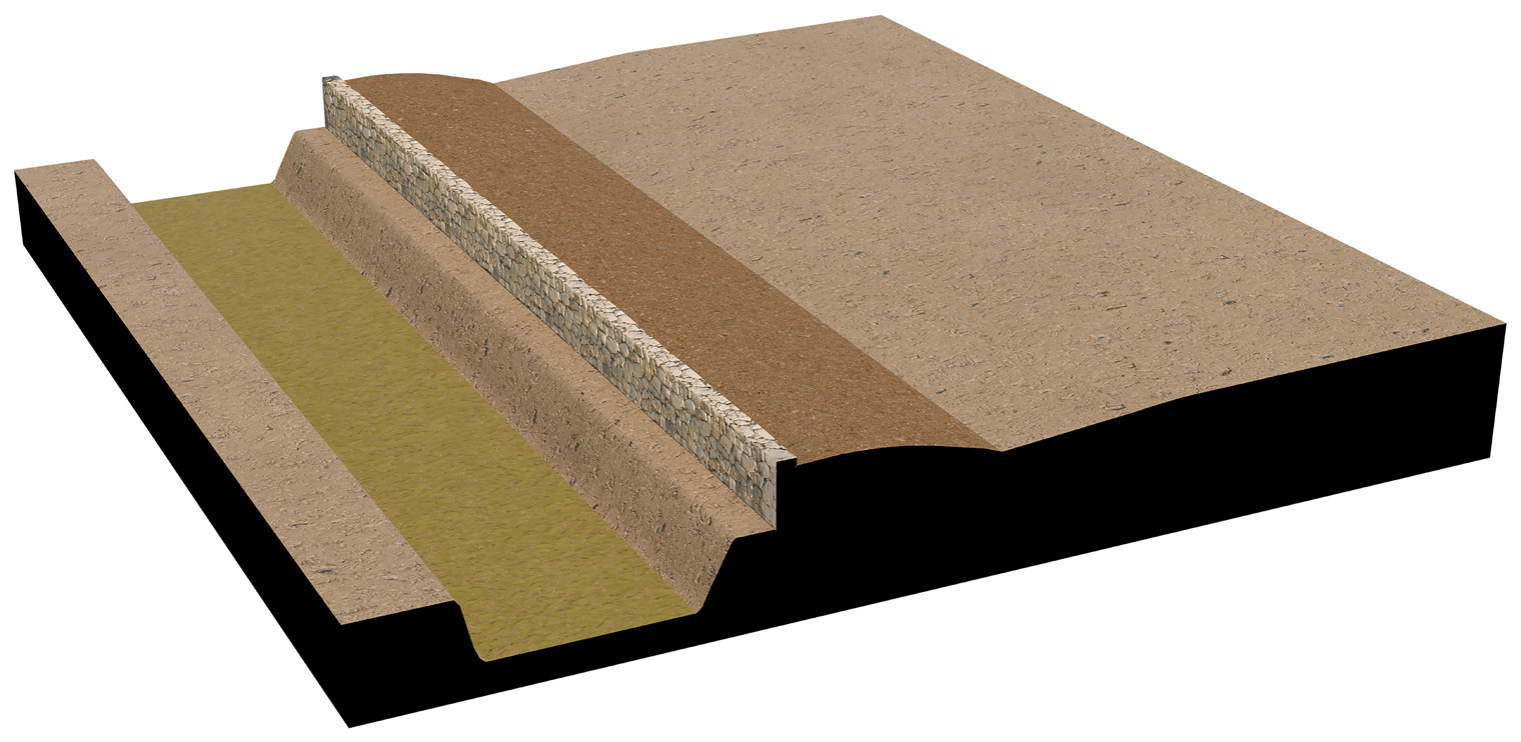
3D model of the agger wall construction technique.

The agger-wall system was modelled using traditional manual techniques, as applying BIM to non-building structures remains challenging. The semantic structure of BIM, in fact, is not inherently flexible and is typically not designed to accommodate features beyond architectural buildings. The model represents the first phase of the fortification, dated to the early 7th century BC, in which the embankment was stabilised by a stone retaining wall on the outer side, facing the ditch. This reconstruction was intended to clearly illustrate the construction technique, and thus only a small segment of the landscape was rendered in three dimensions. All available measurements were derived from excavation plans and sections, while some aspects—such as the total height of the wall—are based on plausible hypotheses (see Figure 3).

**Figure 4.**
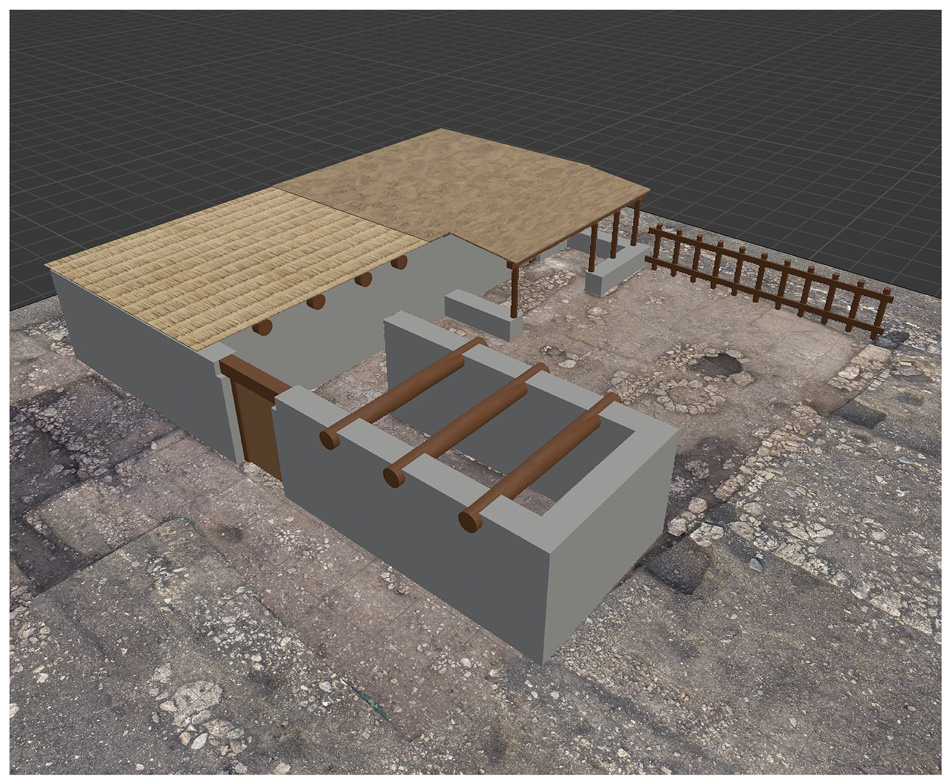
BIM model of house 113W4, with different roof layers (from left to right): wooden beams, reed matting, and raw earth.

For the second case study—the house on plot 113W4—the modelling process began with the import of relevant excavation data into Blender, specifically an orthophoto of the final excavation phase, which was scaled to match the software’s unit system. Using Bonsai, a new IFC project was created, and a spatial instance of the building was defined. Within the semantic structure of BIM, this instance functions as a "spatial container" that houses all associated construction elements. These components were defined using standard IFC classes ^13^, and their geometry was modelled based on both estimated measurements and established knowledge of construction techniques prevalent in the 7th century BC. One of the primary challenges during the modelling process was the accurate reconstruction of the beam layout used to support the roof. In this context, the virtual reconstruction proved essential for testing and verifying the feasibility of multiple structural hypotheses. Information regarding the materials used in construction was also integrated into the model. The building materials correspond to those locally available in the area during the period, including stone, wood, and perishable organic components. Upon completion, the BIM model of house 113W4 was used to estimate the volume of materials required for its construction—data that can serve as a basis for more precise architectural energetics calculations (see Figure 4).

## Architectural energetics calculations

### The ditch and the house

Various methods exist for estimating the costs of ancient construction projects. Greek and Latin epigraphic sources provide detailed building accounts, listing expenses in local currencies, but these records pertain exclusively to monumental and public structures, particularly temples. For domestic architecture, we must turn to architectural energetics—a methodology developed primarily in Mesoamerican archaeology but later applied to Mediterranean contexts, where epigraphic building accounts serve as useful comparative data ^14^. The principle of architectural energetics involves breaking down a building project into separate but interconnected tasks, each requiring a measurable labour investment, expressed in person-days (p-d). In ancient construction, the key tasks included raw material procurement, transportation, and assembly. By quantifying these steps, we can estimate the total labour required to construct a house from scratch, providing valuable insight into the broader effort needed to build sufficient housing for an ancient community.

In the following charts, we present architectural energetics calculations for construction of the agger-wall and of the house on plot 113W4. This analysis is based on a substantial body of scholarship from the 1990’s, in which the methodology was applied across various architectural contexts and building programs (see note 14). However, adapting these results to the present study required careful selection and adjustment of figures ^15^. One key modification concerns the definition of a working day. Like most scholars, we assume a fixed daily labour duration, accounting also for seasonal variations—shorter daylight hours in winter and extreme heat in summer, which limited effective working time. However, given that these were predominantly agrarian societies, labourers engaged in construction would still have had other essential duties, particularly in food production. We take these duties and we therefore define an effective working day as eight hours—a practical year-round average for outdoor labour. Beyond labour time, several site-specific factors influence construction costs. Access to raw materials varies depending on the local environment, significantly affecting transportation costs. Additionally, workforce availability differed between cities and must be adjusted accordingly—this aspect will be addressed further in the paper.

The architectural energetics calculations for the agger-wall are described and presented in the following chart. The rather simple architecture of this fortification requires no additional explanations (see Table 1; also Figure 3).

**Table 1.** Architectural energetics calculations for the 7th century BC agger-wall in Megara Hyblaia.

The agger-wall	Person-days (p-d)	Comments ^16^
Ditch Digging		Width = 7 m; Depth = 2 m; Length = 1500 m Total volume = 21000 m3
soft levels	436,8	Depth = 0,4 m; Volume = 4200 m3
mixed levels	514,5	Depth = 0,2 m; Volume = 2100 m3
bedrock	5108,25	Depth = 1,4 m; Volume = 14700 m3
Removal		
loose rubbles	567	Volume = 6300 m3
rubble stones	661,5	Volume = 1800 m3
Fortification Construction		Length = 1500 m
bank	1039,5	Width = 3,6 m; Height = 2 m; Volume = 10800 m3
wall	2250	Width = 0,6 m; Height = 2 m; Volume = 1800 m3
finishing	900	External wall facing = 3000 m ^2^
**TOTAL**	**11477,55**	

The house on plot 113W4, however, needs some contextualisation. All excavated archaic houses in Megara feature full-stone walls. Ground plans evolved from single-square-room houses in the late 8th century to the pastas house, which—with minor variations—became the canonical layout from the early 7th century BC onward. While plot sizes varied across different sectors, an average of approximately 120 m ^2^ can be established. Two scenarios for the procurement of stone are proposed here below. Scenario 1: the stone was extracted *ad hoc*. Scenario 2: the construction stone (rubble stones) came from the material extracted when the ditch was dug ^17^. Both scenarios are discussed hereafter (see Table 2; also Figure 4).

**Table 2.** Architectural energetics calculations for the 7th century BC house on plot 113W4 in Megara Hyblaia.

House on plot 113W4	Person-days (p-d)	Comments
Stone (walls)		Volume = 44,29 m3
scenario 1: extraction ^19^	28,79	
scenario 1: transport	25,3	On back of men ^20^
scenario 2: transport	63,27	On back of men ^21^
Wood		
beams, door, door frame ^22^	27,11	Volume = 4,28 m3
east side enclosure	5,11	Volume = 0,73 m3
pastas props and beams	2,92	Volume = 0,46 m3
transport	5,25	One oxen yoke, with driver ^23^
loading/unloading carts ^24^	0,82	
Foundations levelling ^25^	0,44	Depth = 0,05 m; Volume = 1,26 m3
Walls construction ^26^	55,36	
Backfilling + floor foundation ^27^	1,18	Thickness = 0,1 m; volume = 12,3 m3
Roofing (framework) ^28^	8,38	
Pastas		
wood processing ^29^	0,61	Squaring, cutting
posts setting ^30^	1,25	
roof framework ^31^	0,625	
Roofing (flat roofs) ^32^	8,71	Surface = 85,42
Openings		
wood processing	2,79	Squaring, cutting
door construction ^33^	4	
door and doorframe setting ^34^	3	
Enclosure		
wood processing	3,02	Squaring, cutting
construction	2	
**TOTAL (scenario 1)**	**186,67**	
**TOTAL (scenario 2)**	**195,85**	

At first glance, scenario 1 seems more advantageous in that it would cost 9 p-d less to construct the house. This is largely due to the nearness of the raw material, as we consider here that it came from the cliffs topping the river Cantera, some 100 m away ^18^ (the nearest sections of the ditch being some 250 m away). Yet, it would seem reasonable to assume that the ditch material was reused somehow, as it provided thousands of cubic metres of already extracted stone (see note 17). Besides, while the local bedrock could conveniently be used for the few first 8th-century houses (around 15 m ^3^ of stone each), it appears problematic to locally extract the 4,429 m ^3^ required for a hundred 7th-century houses, like the one on plot 113W4, thus leaving several parts of the urban space with large diggings. Eventually, the best-case scenario would involve the transport of stone from the ditch by carts pulled by oxen: in this case, taking into account the loading/unloading of the carts, it would cost some 11.27 p-d (and 7.96 oxen-days). The house on plot 113W4 could thus be built in 143.85 p-d. Such a scenario would nevertheless imply the use of oxen yokes, which makes perfect sense in the case of the long wooden logs taken some 7 km away from the construction site, but seems less convenient here.

### Demography and workforce in early 7 ^th^ century Megara

Estimating workforce availability in early construction projects is particularly challenging, especially for early periods such as archaic Sicily. The figures presented here can only be properly interpreted within the broader context of ancient demography and the resulting labour force capacity. The time period here considered are the first decades of the 7th century BC in Sicily, namely the time of the second generation of settlers, roughly 50 years after the foundation. Assessing the impact of construction programs on a community depends entirely on the number of individuals available for labour—the implications differ significantly if 100 people, rather than 1,000, could be allocated to building activities. Moreover, as previously noted, agricultural cycles played a critical role in workforce distribution. Certain periods of the year were dominated by food production, while other essential tasks—such as the manufacture of pottery, textiles, and furniture—demanded year-round attention (for instance, Brysbaert, 2023). To accurately estimate the available labour force, it is therefore essential to consider the agricultural calendar and seasonal labour constraints. Various demographic estimates for ancient Mediterranean populations have been proposed, often yielding significant variations. Although inherently theoretical, even when supplemented with archaeological data, the models developed by M.H. Hansen within the Copenhagen Polis Centre remain the most comprehensive ^35^. Following Hansen’s methodology, we would have a 7200–9600 population figure for Megara Hyblaia ^36^. Nevertheless, these are only rough average mathematical estimates, which may only correspond to a certain period of each city’s urban development, namely the urban development’s heyday. They surely cannot apply to the first and even second generations contexts, as defined here for our purpose. We use a different approach here, based on: population estimates in Megara by the late 6 ^th^/early 5 ^th^ c. BC, population estimates upon foundation time and a fixed annual growth ^37^. The following results are presented on two lines corresponding to the two possible population estimates at the beginning (see Table 3).

**Table 3.** Population estimates (min and max) at different periods in Megara Hyblaia.

	Years			
	0 (foundation)	+25 (1 ^st^ generation)	+50 (2 ^nd^ generation)	+200 (late 6 ^th^ c.)
Population	150	246	**404**	7873
	200	328	**538**	10497

Demography, however, serves only as a baseline for estimating potential workforce availability. Not all members of a community could engage in the physically demanding tasks of construction. Nonetheless, we challenge the conventional assumption that only able-bodied adult males (aged 18–50) participated. These estimates are thus generally based on army figures, then adapted to workforce by taking into account the wealthiest classes which were probably exempted from these duties, resulting in a 22,5% of the whole population ^38^. While activities such as stone extraction or heavy transport were likely performed by adult men, women and older adolescents could have contributed to other construction-related duties ( Turner, 2021). Based on these assumptions, we will rather consider a workforce average of 50% of the total population ^39^ hence, after Table 3, between 202 and 269 people.

Finally, ancient agricultural practices are well-documented in Hesiod’s writings, which provide invaluable insight into the daily rhythms of agrarian communities in the 8th-century BC Greek world. This unique source allows for the reconstruction of a realistic agricultural calendar, helping to determine periods of workforce availability ( Brysbaert, 2023; Fitzjohn, 2013; Ingold, 1993). Based on this framework, we estimate that labourers could be available for 150–180 days per year, corresponding to 5–6 months during which they were relatively free from other obligations. From the previous figures, we can deduce that the total of person-days under the worst-case scenario (lowest population x lowest availability) is 30300 and under the best-case scenario (highest population x highest availability) is 48420.

Dividing the number of person-days available yearly for construction by the energy expenditures required to build a house yields the number of new houses that could be constructed within a year (under both worst-case and best-case scenarios). Then, assuming an average household size of five people, we can estimate the number of individuals that these newly built houses could accommodate ^40^ (see Table 4).

**Table 4.** Number of new houses and their occupants (min and max), during the 2 ^nd^ generation in Megara Hyblaia.

New houses	146
	233
Occupants (persons)	730
	1165

## Conclusions

This short essay sets out to assess, for the first time, the feasibility of two critical and interrelated construction projects undertaken by an early Greek Sicilian community. In doing so, we have remained acutely aware of the limitations and uncertainties inherent in both architectural energetics calculations and ancient demographic estimates. Taking these constraints into account—and with due caution—our findings suggest that the second generation of inhabitants at Megara Hyblaia, living approximately in the first quarter of the 7th century BC, possessed sufficient human resources to construct both the initial fortification and an adequate number of houses to accommodate the entire population. Notably, these foundational undertakings for communal development could likely have been completed within a single year, without compromising agricultural production or the performance of other essential tasks. Future research will need to refine estimates of both energetic expenditures and demographic structures, in order to more precisely evaluate the costs and labour demands associated with each aspect of the construction programmes. These analyses should also extend to include civic and religious architecture, as well as critical infrastructure such as roads and water management systems. To provide a more comprehensive picture, the inclusion of food and artifact production is essential. Finally, it would be valuable to apply this methodology to other significant periods in the history of Megara Hyblaia—particularly the 6th century BC, when major construction projects such as temples, civic buildings, and fortification walls were undertaken. This later phase should be examined with reference to its evolving sociopolitical context, marked by a more structured society and the emergence of labour specialisation

Besides, this study has demonstrated the potential of 3D modelling and BIM tools in the analysis of ancient architectural structures, despite certain known limitations. While BIM’s defined framework offers significant advantages, it can struggle to accommodate the complexity and irregularity of heritage subjects, especially non-standard elements like the agger-wall. Nevertheless, the capacity of these tools to integrate visualization, analysis, and data makes them highly valuable. Future developments will focus on incorporating 4D and 5D modelling into the models, generating automated estimations of construction time and cost based on detailed input data. In this way, it will be possible to further enhance the scope and utility of digital heritage models.

## Data Availability

The data that support the findings of this study are openly available: In Datarepository Unive (Ca' Foscari University of Venice) At https://doi.org/10.71731/DATA_UNIVE/XU7LS1 ( MeGE, 2025) Data are available under the terms of the Attribution-NonCommercial-NoDerivatives 4.0 International (CC BY-NC-ND 4.0).
