# Supplementary material for: The Ditch and the House. Construction Energetics in Early 7th-century BC Megara Hyblaia: A Preliminary Study
Source: Open Res Eur. 2025 Dec 3;5:184. Originally published 2025 Jul 14. [Version 2] doi: 10.12688/openreseurope.20518.2 (PMC12754354; doi:10.12688/openreseurope.20518.2)
Supplement: Peer review history

# Reviewer report: Reviewer response for version 2

**DOI:** 10.21956/openreseurope.23598.r65629

**Published:** 2026-01-06

**Authors:** İrfan Kocaman

**Affiliations:** 1 Erzurum Technical University, Erzurum, Turkey

**Reviewer Comments**

 The manuscript presents an original and interdisciplinary approach to evaluating early construction capacity through architectural energetics supported by 3D modelling and BIM-based volumetric estimations. The study is clearly structured, well-documented archaeologically, and contributes valuable quantitative insight into early Archaic urban development. Overall, the paper is suitable for publication after **minor revisions** aimed at improving clarity and methodological transparency, particularly from an engineering-oriented perspective.

1. While the architectural energetics framework is well applied, it would be beneficial to more explicitly clarify that the proposed dimensions of the agger-wall (e.g., wall thickness and height) are interpretative reconstructions rather than performance-based engineering designs. A short clarification acknowledging this distinction would help avoid possible misinterpretations by readers from engineering disciplines.
2. The discussion of construction materials is archaeologically sound; however, briefly indicating typical ranges of material properties reported in the literature (e.g., for dry rubble masonry or local stone) would strengthen the transparency of the assumptions without requiring additional analyses.
3. The use of BIM as a tool for geometric reconstruction and volumetric estimation is well justified. It may be helpful to explicitly state that BIM is employed here primarily as a quantitative modelling aid rather than for structural or performance-based analysis, in order to clearly frame its methodological role.
4. The labour and productivity rates adopted are reasonable and well referenced. A short statement acknowledging that these values represent average estimates—and that minor variations would not significantly affect the overall conclusions—would further reinforce the robustness of the results.
5. The conclusions are coherent and well aligned with the presented data. A brief reminder that the findings represent a feasible scenario under favourable conditions would appropriately contextualize the results while preserving their significance.

The paper is scientifically sound, methodologically innovative, and clearly written. The suggested revisions are minor and primarily intended to enhance clarity and interdisciplinary readability rather than to request additional analyses. After these small adjustments, the manuscript will be well suited for publication.

If any, are all the source data and materials underlying the results available?

Yes

Is the work original in terms of material and argument?

Yes

Is the work clearly and cogently presented?

Yes

Is the argument persuasive and supported by evidence?

Yes

Does the research article contribute to the cultural, historical, social understanding of the field?

Yes

Does it sufficiently engage with relevant methodologies and secondary literature on the topic?

Yes

Reviewer Expertise:

Structural and earthquake engineering; seismic performance assessment of masonry and historical structures; numerical modelling (FEM); dynamic analysis of heritage buildings; strengthening and conservation of cultural heritage structures.

I confirm that I have read this submission and believe that I have an appropriate level of expertise to confirm that it is of an acceptable scientific standard.

# Reviewer report: Reviewer response for version 2

**DOI:** 10.21956/openreseurope.23598.r65633

**Published:** 2025-12-30

**Authors:** Ifechukwu Gil-Ozoudeh

**Affiliations:** 1 Enugu State University of Science and Technology, Enugu, Enugu, Nigeria

The manuscript explores how digitalisation and intelligent technologies—such as BIM, IoT, AI, VR, and smart systems—are transforming environmental design. It presents a broad literature review and a detailed case study of Chongqing Lijia Smart Park, using hierarchical analysis and fuzzy evaluation to assess the effectiveness of digital-intelligent integration in environmental design. The article concludes with projected trends, including human-centred design, sustainability, interdisciplinary integration, and enhanced user participation.

 Overall, the topic is timely and relevant, especially in the context of smart cities and data-driven environmental design. The case study is well chosen and aligns with the article’s objectives.

**Major Comments (Points that Must Be Addressed)**

**1. Originality of the Argument — *Partly***

 While the manuscript covers important themes, its originality is moderate. Much of the discussion reiterates established knowledge (e.g., BIM benefits, IoT sensing, AI-enhanced UX). The article would benefit from a clearer statement of **what is new** about the proposed “innovation paths.”

**Required Improvement:**

 Add a distinct contribution statement clarifying what novel insight or framework the paper adds beyond existing literature.

**2. Engagement with Secondary Literature — *Partly***

 The literature cited is extensive but not always critically synthesised. The review occasionally becomes descriptive rather than analytical.

**Required Improvement:**

 Strengthen the literature engagement by comparing differing scholarly perspectives, identifying gaps, and highlighting where the authors’ argument intervenes.

**3. Methodological Rigour — *Partly***

 The hierarchical analysis + fuzzy evaluation approach is sound, but more methodological transparency is needed.

**Must Clarify:**

- How the evaluation indicators were selected and validated
- The criteria for expert selection
- Potential biases in the questionnaire (sampling, demographics, representativeness)
- Limitations of a single-site case study

Without these clarifications, the robustness of the findings remains uncertain.

**4. Clarity and Coherence — *Partly***

 The manuscript is readable but contains:

- Long, repetitive sentences
- Occasional grammatical inconsistencies
- Paragraphs with overlapping ideas
- A need for clearer transitions between major sections

**Required Improvement:**

 A full language and structure polishing is recommended. Streamline repeated content in the introduction and discussion.

**5. Persuasiveness of the Argument — *Partly***

 The empirical results are well presented but the analysis is somewhat descriptive.

**To strengthen persuasiveness, please:**

- Explain *why* certain indicators performed better or worse
- Discuss unexpected findings
- Connect the results more explicitly to the theoretical claims
- Reflect on how the findings advance environmental design practice as a discipline

**Minor Comments (Recommended Enhancements)**

1. Consider adding diagrams or conceptual figures summarising:
  - The integration framework of digital + intelligent + environmental design
  - The evaluation model
2. The conclusion would be stronger with:
  - A clearer articulation of global relevance
  - More specific future research directions
3. Some references appear region-specific; include more international comparative works if possible.
4. Ensure consistent terminology (e.g., “environmental design,” “environment design,” “environmental art design”).

**Overall Assessment**

 The manuscript addresses an important topic and demonstrates significant effort in both theoretical synthesis and empirical evaluation. However, revisions are needed to improve the originality, analytical depth, clarity of methodology, and coherence of argumentation.

**My recommendation:**

**Revision Required (Moderate to Minor)**

 Once the issues above are addressed, the article has the potential to make a meaningful contribution to discussions on digital and intelligent transformations in environmental design.

If any, are all the source data and materials underlying the results available?

Yes

Is the work original in terms of material and argument?

Partly

Is the work clearly and cogently presented?

Partly

Is the argument persuasive and supported by evidence?

Partly

Does the research article contribute to the cultural, historical, social understanding of the field?

Partly

Does it sufficiently engage with relevant methodologies and secondary literature on the topic?

Partly

Reviewer Expertise:

Architecture, Renewable Energy, Sustainability Urban Housing, Materials Engineering

I confirm that I have read this submission and believe that I have an appropriate level of expertise to confirm that it is of an acceptable scientific standard, however I have significant reservations, as outlined above.

# Reviewer report: Reviewer response for version 1

**DOI:** 10.21956/openreseurope.22202.r60915

**Published:** 2025-10-14

**Authors:** Vasiliki Palieraki

**Affiliations:** 1 Civil Engineering, National Technical University of Athens Ship Design Lab, Zografou, Attica, Greece

The current study is very interesting. Adding some additional information regarding the research, could be helpful for the readers.

 1. In page 5, in the paragraph "3D modelling workflow" you are writing about the information regarding the materials. You are suggested to add some information in the paper, regarding the materials as well as the height of the remainings.

 2. You are suggested to describe in more details the model shown in Figure 4, and based on which information the model has been constructed.

 3. According to the reviewer, the width of the wall (0,6m) seems rather limited, while on the other hand the height, in case it refers to the initial height, is again small for such a structure.

 4. Please, explain how the walls have been constructed, and if there is information about the use of mortar and not only stones.

If any, are all the source data and materials underlying the results available?

No source data required

Is the work original in terms of material and argument?

Yes

Is the work clearly and cogently presented?

Partly

Is the argument persuasive and supported by evidence?

Partly

Does the research article contribute to the cultural, historical, social understanding of the field?

Yes

Does it sufficiently engage with relevant methodologies and secondary literature on the topic?

Yes

Reviewer Expertise:

Civil Engineering, Ancient Monuments, Behavior of monuments, experimental testing, analysis

I confirm that I have read this submission and believe that I have an appropriate level of expertise to confirm that it is of an acceptable scientific standard.

# Response

**Published:** 2025-11-04

**Authors:** Frédéric Mège

**Affiliations:** Department of Humanities, Ca' Foscari University Department of Humanities, Venice, Veneto, Italy

Dear colleague, Thank you for taking the time to read our article and for your valuable comments. Your remarks have certainly helped improve the quality of our study. Most of your comments (points 1, 2, and 4) have been taken into account and had, in fact, also been raised by the first reviewer, Felat Dursun. Regarding point 3 specifically, we understand that the dimensions of the wall may appear somewhat limited from a defensive perspective. However, this type of wall formed only part of a broader system: it was preceded by a large ditch and followed by a relatively wide embankment. Moreover, such a structure also had a symbolic purpose: to delineate the boundaries of the urban space (asty) in relation to the suburb (proasteion) and the territory (chôra), while signalling to outsiders the presence of a “civilised” settlement. Indeed, after some fifty years, the Megarians felt the need to construct a more substantial fortification built with large ashlar blocks (2nd and 3rd phases). As for the dimensions, we deduced the wall’s width (0,6 m) from sections drawn in the poorly preserved southern and eastern parts of the agger-wall, while the 2 m height is an informed estimate based on the extant remains (around 1,5 m) and on the likelihood that the wall was built without scaffolding, as was the case for domestic walls, on such an early period. We hope that we accordingly answered to your questioning and we would like to thank you again for your review.

 With best regards, Frédéric Mège and Eleonora Delpozzo.

# Reviewer report: Reviewer response for version 1

**DOI:** 10.21956/openreseurope.22202.r57321

**Published:** 2025-09-01

**Authors:** Felat DURSUN

**Affiliations:** 1 Department of Conservation and Restoration of Cultural Heritage, Izmir Institute of Technology, Urla,, İzmir, Turkey

**Review on**

**The Ditch and the House. Megara Hyblaia by the early 7th c. BC.**

 This study aims to assess whether the Megarian community of the period had the workforce and time needed to meet the costs of the construction projects under consideration. For this purpose, the authors focused on Architectural Energetics calculations for two case studies: the agger-wall fortification and the house located on plot 113W4. The article provides useful data and serves as an important example of applying Architectural Energetics. However, the basis and accuracy of the calculations, as well as the integration of BIM applications, should be supported with more concrete evidence. Adding visual materials would help readers better understand the site and link the interpretations to the context. It would also be valuable to compare the results with similar studies to give a wider perspective and highlight the importance of the findings.

 Below the author can follow my notes and recommendations,

 Considering the title of the manuscript, it is recommended to make it more specific and revise it so that it clearly reflects the aim and focus of the study. In its current form, the title does not adequately reflect the concept or purpose of the research.

**Abstract:**

 The structural format of the abstract is generally acceptable; however, it is recommended to revise the results and conclusion subsections so that the results focus on the actual findings rather than interpretations (particularly in the results section). the conclusion section, on the other hand, some points are repeated from the introduction, which should be avoided. Instead, it would be more effective to emphasize the core results and the main conclusions of the study.

**Intro:**

 -please provide supportive images of the study area, overall view, the mentioned ditch, the dimensions of the stone, and agger-wall, etc.

 -please provide additional references, especially on reconstruction of ancient lands and the use of local materials in ancient dwellings. (see p4: 3D modelling and BIM for archaeological reconstructions)

 -Figure 1. Schematic plan of archaic Megara Hyblaia.

 Here it would be better to provide some images from the site to grasp the overall view of the site.

 -Table 1. Architectural energetics calculations for the 7th century BC agger-wall in

 Megara Hyblaia.

 Please be more specific regarding the bases of your estimated person-days calculations

**Architectural energetics calculations**

 -Regarding the following statements: (p7)

 Statement1: “Yet, it would seem reasonable to assume that the ditch material was reused somehow, as it provided thousands of cubic metres of already extracted stone (see note 17).”

 To reach such a statement it is recommended to evaluate the stone layers. without assessing the discontinuity sets and their relationship to the bedding plane, thickness, etc. it is not possible to conclude whether a stone layer is suitable for cut stone production. A proper building stone suitability assessment combines discontinuity analysis (often done via scanline surveys or mapping), laboratory testing, and quarrying feasibility studies. yes, they can be used as aggregate for mortar and plaster production, however as a cut stone, it is essential to evaluate their block size and shape. for insantce, if discontinuities are too closely spaced, the stone will break into small fragments rather than yielding large blocks. If they are too far apart or poorly oriented, the extraction process becomes costly or impossible.

 Statement2: “Scenario 1: the stone was extracted ad hoc. Scenario 2: the construction stone (rubble stones) came from the material extracted when the ditch was dug. (P7-8)”

 In both scenarios, the approach applied in the calculations appears open to discussion. Esp., the observations in Scenario 1 involve considerable uncertainties. Could you please clarify the rationale for assuming that the raw material originated from cliff toppings and indicate the basis for this assumption? If any quarry has been identified, it would be helpful to provide supporting explanations and relevant visual documentation.

 - The discussion of the results, particularly in relation to studies conducted in the close vicinity or within a similar archaeological period, is lacking. Including comparisons with such studies would strengthen the discussion and provide valuable context for the findings.

**Conclusions**

 Here, there is a strong emphasis on the study’s limitations and suggestions for further research. When considered within the framework of Architectural Energetics, this “short essay” lacks certain detailed analyses and findings that could significantly influence the calculations. However, as the present study holds substantial value on its own, it may be beneficial to characterize it as a preliminary study.

If any, are all the source data and materials underlying the results available?

No source data required

Is the work original in terms of material and argument?

Yes

Is the work clearly and cogently presented?

Partly

Is the argument persuasive and supported by evidence?

Partly

Does the research article contribute to the cultural, historical, social understanding of the field?

Yes

Does it sufficiently engage with relevant methodologies and secondary literature on the topic?

Partly

Reviewer Expertise:

stone conservation, geoheritage, geoarchaeology, material characterization, site selection

I confirm that I have read this submission and believe that I have an appropriate level of expertise to confirm that it is of an acceptable scientific standard, however I have significant reservations, as outlined above.

# Response

**Published:** 2025-11-04

**Authors:** Frédéric Mège

**Affiliations:** Department of Humanities, Ca' Foscari University Department of Humanities, Venice, Veneto, Italy

Dear colleague, Thank you very much for taking the time to read our article and for providing your valuable comments. Your remarks have certainly helped us improve the quality of our study. We would nevertheless like to respond to some of your points and explain how we have addressed them in the revised version. As you rightly suggested, the title of the manuscript has been modified accordingly. In addition, the *Results* section of the abstract now lists only the main findings, as is appropriate. Following your recommendation, we have added three new figures to better support the text and help readers visualize the site and the structures under investigation. The calculations in Table 1 are now described in greater detail, which was indeed missing in the original version. Finally, as per your suggestion, the architectural energetics results for the house are now compared with similar data, allowing readers to place our findings in a broader context. Regarding the construction materials in general, and stone in particular, we have worked to clarify our hypotheses and the evidence on which they are based. However, as often happens in Classical archaeology, the type of detailed data you requested—such as fine characterisations determining the suitability of specific stones for construction—are unfortunately lacking. One of us has, however, carried out a separate project that included petrographic analyses of the main stones used in Megara, and some of these results are now incorporated into the article. It should be emphasized that the stones used for the retaining wall of the agger-wall and the house were small ashlars, possibly roughly dressed, or even rubble stones. Neither of these structures employed the large ashlar blocks used in Megara’s monumental buildings (such as the second and third phases of the Archaic fortifications, temples, and civic buildings of the late 7th and especially 6th centuries BC). Those large blocks were extracted from more distant quarries that provided suitable stone for major construction projects. Therefore, we have maintained our initial hypothesis—supported by geological observations and investigations—that the rubble stones used for the 7th-century fortifications and houses were locally sourced, in some cases simply collected. The reuse of material extracted from the bedrock during ditch excavation to build houses remains, in our view, highly plausible. The cliffs bordering the plateaus where the urban area was located also appear to have been suitable places from which builders could obtain the rough ashlars they required. We hope that these clarifications and the revisions made to the article will help you gain a clearer understanding of our study. Once again, we sincerely thank you for your time and for your thoughtful and constructive feedback. With best regards, Frédéric Mège and Eleonora Delpozzo.
